# Consistency of small‐field dosimetry, on and off axis, in beam‐matched linacs used for stereotactic radiosurgery

**DOI:** 10.1002/acm2.13160

**Published:** 2021-01-13

**Authors:** Luis Muñoz, Tomas Kron, Marco Petasecca, Joseph Bucci, Michael Jackson, Peter Metcalfe, Anatoly B. Rosenfeld, Giordano Biasi

**Affiliations:** ^1^ Genesiscare Flinders Private Hospital Bedford Park SA Australia; ^2^ Centre for Medical Radiation Physics University of Wollongong NSW Australia; ^3^ Peter MacCallum Cancer Centre Melbourne VIC Australia; ^4^ St. George Cancer Care Centre St George Hospital Kogarah NSW Australia; ^5^ Genesiscare Waratah Private Hospital Hurstville NSW Australia; ^6^ University of New South Wales Kensington NSW Australia

**Keywords:** Elekta, matched linacs, off‐axis, small‐field dosimetry, SRS

## Abstract

**Purpose:**

Stereotactic radiosurgery (SRS) can be delivered with a standard linear accelerator (linac). At institutions having more than one linac, beam matching is common practice. In the literature, there are indications that machine central axis (CAX) matching for broad fields does not guarantee matching of small fields with side ≤2 cm. There is no indication on how matching for broad fields on axis translates to matching small fields off axis. These are of interest to multitarget single‐isocenter (MTSI) SRS planning and the present work addresses that gap in the literature.

**Methods:**

We used 6 MV flattening filter free (FFF) beams from four Elekta VersaHD® linacs equipped with an Agility™ multileaf collimator (MLC). The linacs were strictly matched for broad fields on CAX. We compared output factors (OPFs) and effective field size, measured concurrently using a novel 2D solid‐state dosimeter “Duo” with a spatial resolution of 0.2 mm, in square and rectangular static fields with sides from 0.5 to 2 cm, either on axis or away from it by 5 to 15 cm.

**Results:**

Among the four linacs, OPF for fields ≥1 × 1 cm^2^ ranged 1.3% on CAX, whereas off axis a maximum range of 1.9% was observed at 15 cm. A larger variability in OPF was noted for the 0.5 × 0.5 cm^2^ field, with a range of 5.9% on CAX, which improved to a maximum of 2.3% moving off axis. Two linacs showed greater consistency with a range of 1.4% on CAX and 2.2% at 15 cm off axis. Between linacs, the effective field size varied by <0.04 cm in most cases, both on and off axis. Tighter matching was observed for linacs with a similar focal spot position.

**Conclusions:**

Verification of small‐field consistency for matched linacs used for SRS is an important task for dosimetric validation. A significant benefit of concurrent measurement of field size and OPF allowed for a comprehensive assessment using a novel diode array. Our study showed the four linacs, strictly matched for broad fields on CAX, were still matched down to a field size of 1 x 1 cm^2^ on and off axis.

## 1 INTRODUCTION

In stereotactic radiosurgery (SRS), small highly modulated beams are used to deliver ablative doses to brain metastases in single or hypo‐fractionated regimens.^1^ Stereotactic radiosurgery is an established treatment for patients with one to four metastases.^2^, ^3^, ^4^ Prospective and retrospective studies have reported on its feasibility and clinical benefit also for patients with ≥5 metastases.^5^, ^6^, ^7^

Stereotactic radiosurgery can be delivered with a standard C‐arm linear accelerator (linac) equipped with a high‐definition multileaf collimator (HD‐MLC), a 6 degree‐of‐freedom (DoF) robotic treatment couch,^8^ and a system for image guidance. For patients with more than one metastasis, treatment cost and time can be minimized with multitarget single‐isocenter (MTSI) planning,^9^, ^10^, ^11^ where the isocenter is placed between targets. Targets can range from small (0.7 cc) to larger volumes (30 cc),^12^ and can be located up to 10 cm from the isocenter.^13^

At institutions with more than one linac, beam matching^14^ is common practice to optimize and better utilize clinical resources. A cohort of matched linacs can be described by the same beam model in the treatment planning system (TPS), so that pretreatment quality assurance (QA) and treatment delivery can be performed on any of those linacs. The matching procedure typically considers only broad square fields (10 × 10 cm^2^ to 30 × 30 cm^2^) centered on machine central axis (CAX), with linac gantry and collimators at angle 0°. It requires that, across the linac cohort, percentage depth dose (PDD) at 100 cm source to surface distance (SSD) be in agreement to within ±1% or less, and that point measurements in the specified range of a lateral dose distribution be in agreement to within ±2%.^15^, ^16^

The matching procedure neglects small fields (of side ≤2 cm), whose dosimetry presents two particular challenges.^17^, ^18^ The first is related to the dosimeter, its positioning, the reproducibility of measurement and the correction factors required to relate its reading to actual dose. These corrections vary as a function of linac design, field size, depth of measurement, and distance from the x‐ray source.^19^ The second is related to the radiation beam, where small variations in effective field size and shape have a strong influence on the output factor (OPF). A sub‐millimeter variation in size for a field 1 cm across can lead to an OPF variation of several percent.^20^, ^21^, ^22^ This would also include consideration of focal spot size and position and subsequent influence on small‐field dosimetry.

In the literature there are a few indications that matching for broad fields on CAX does not guarantee matching in small fields on CAX.^22^, ^23^ At present, there is no indication on how matching for broad fields on CAX translates to small fields delivered off axis, which are of interest to MTSI SRS planning. The present work addressed the gap in the literature by assessing the consistency of small‐field dosimetry in four matched linacs that can be used for MTSI SRS. We considered both fields centered on the machine CAX and away from it by a distance in the range from 5 to 15 cm.

## 2 MATERIALS AND METHODS

### 2.A Linacs

The four linacs (referred to as FS1, ST3, TC1, and TC2) were Elekta Versa HD® (Elekta, Crawley, UK), equipped with Agility™ MLC of leaf width 5 mm. Their 6 MV flattening filter free (FFF) beams were matched using criteria recommended by Rijken et al.,^24^ which add to those outlined by the vendor.

The vendor requires that, in square fields of side 10 and 30 cm, water‐scanned PDD shall be, at 100 cm SSD, within ±1% of baseline, and that, in the area within 80% of full width at half maximum (FWHM) of any lateral dose distribution measured at a depth of 10 and 90 cm SSD, point dose shall be within 2%.^16^, ^25^ Those point‐dose measurements shall be averaged over a 1‐cm range from the point. Rijken et al.^24^ required that, for the same square fields, PDD shall be within ±0.5% of baseline, and that any averaged point dose in the area within 80% of FWHM shall be within ±1%. Couch, collimator, and gantry runouts shall be within 1 mm. Additionally, parameters such as coincidence of kV‐MV isocenter, and of radiation–mechanical isocenter, were ensured to be ≤1 mm as per TG‐142 tolerances for linacs used for SRS,^26^ and average deviation from the radiation isocenter to a ball‐bearing center for all acquired projections was ≤0.75 mm for Winston‐Lutz tests. These criteria, which are also stricter than those suggested by Hrbacek et al.,^16^ Sarkar et al.,^27^ and Xu et al.^28^ were shown to be sufficient for distributive QA and delivery of cases more complex than single‐target SRS, such as stereotactic body radiotherapy (SBRT) to vertebral lesions.^24^

Prior to the present work, an assessment of matching across the four linacs confirmed adherence to criteria established by Rijken et al.^24^ for square fields of size 10 cm and 30 cm. Beam qualities for 6MV FFF were within 0.5% of a tissue phantom ratio at 20 and 10 cm (TPR_20,10_) of 0.667.

### 2.B Measurements

To assess the consistency of small‐field dosimetry in the four matched linacs, we compared OPF and effective field size (EFS), in square and rectangular static fields defined by the MLC in the cross‐plane and by the diaphragms in the in‐plane; the effective field size can deviate from the nominal size.^29^ Fields were of nominal side in the range from 0.5 to 2 cm, at a depth of 10 and 90 cm SSD, and were either on CAX, or away from it by a distance in the range from 5 to 15 cm along the cross‐plane direction. All fields were produced with the linac gantry and collimator at 0°. In all fields, OPF and EFS were measured concurrently using a novel 2D solid‐state dosimeter, the “Duo.”

**Fig. 1:**
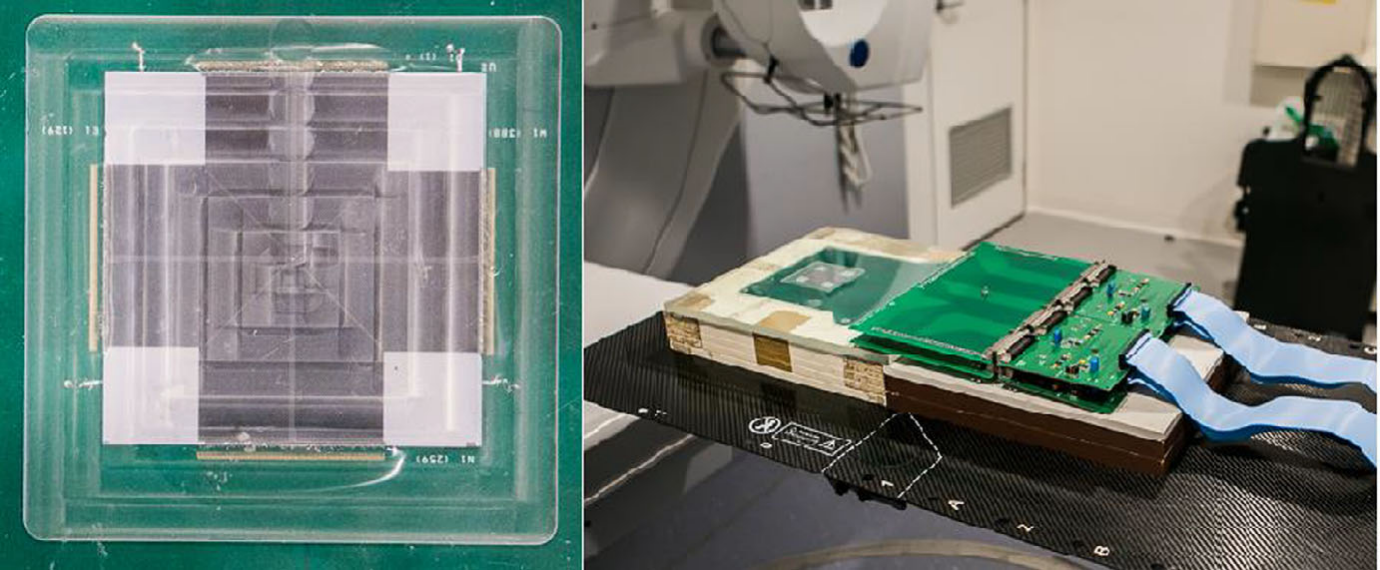
The two‐dimensional active area of the Duo is approximately 5 × 5 cm^2^. 505 diodes (the radiation‐sensitive volumes) are spaced by 0.2 mm on 2 perpendicular linear arrays. In this study, lateral beam profiles were measured along the in‐ and cross‐plane directions, which were defined as the direction of the treatment couch and the direction perpendicular to that, respectively.

The Duo has 505 diodes of area 0.032 mm^2^, spaced by 0.2 mm along two perpendicular linear arrays (Fig. 1). A small air gap on top of the arrays minimizes, in small fields on CAX, the corrections required to relate its readings to dose.^30^ The Duo was used in CIRS Plastic Water (CIRS, Norfolk, VA), and centered on CAX using a square field of side 0.5 cm, by maximizing the response of the central diode. For measurements off axis, subsequent centering was completed by moving the treatment couch, with submillimeter translation shifts from the treatment console using the automatic table movement; the central diode was then assumed as being at the center of the field.

The Duo was demonstrated for small‐field dosimetry on CAX in earlier investigations,^31^, ^32^ which have also discussed limitations and potential. However, the Duo being a novel dosimeter, we chose to complement the present study with a comparative assessment of measurements on CAX using the Duo and two commercial dosimeters: an unshielded diode and film. This assessment was completed on a single linac (FS1).

The unshielded EFD3G (IBA Dosimetry, Germany) diode was paired with a Blue Phantom^2^ (IBA Dosimetry, Germany) water tank and OMNIPRO ACCEPT (IBA Dosimetry, Germany) software. The diode presents with an active volume of 0.19 mm^3^ and requires TRS483 correction for OPF measurement, correcting for its overresponse within small fields.^18^ Centering scans in the water tank were done to ensure diode alignment with the maximum output for each field on CAX, shifts in 0.1 mm increments further verified the position of maximum output. Gafchromic EBT3 films (Ashland, KY, USA) were exposed in CIRS (CIRS Inc., VA, USA) Plastic Water.

### 2.C Data analysis and uncertainties

In this study, because we measured both square and rectangular fields, we reported all results in terms of the equivalent field size (S_eq_), which we defined using the geometric mean:

$$S_{\mathrm{eq}}=\sqrt{A\times B}$$

where A and B were the in‐plane and cross‐plane FWHM, respectively.^18^, ^33^ The same definition was used on and off CAX.

When using the Duo, we applied an equalization procedure to adjust for small differences in the sensitivity of each diode in the arrays.^34^ In any given field, measurements were repeated at least six times, and then the response was taken as the mean of the sample. To determine OPF, the maximum of the lateral profile was used to calculate the detector‐reading ratio, which was reported as a % of the reading in the nominal square field of side 10 cm on CAX. For off axis measurements, the central diode was used to determine the detector‐reading ratio, and not the maximum associated with the inherent asymmetry of the field, and the nominal square field of side 10 cm on CAX was used as the reference field. Supported by earlier investigations,^30^, ^31^, ^32^, ^35^ we did not apply any correction factors to detector‐reading ratios, hereafter referred to simply as OPF, which we reported with an associated accuracy given by the standard deviation of the mean (k = 3), and propagating the error. Lateral beam profiles were analyzed with MATLAB (MathWorks) using a shape‐preserving interpolant function. In that case, measurement accuracy was taken as ±0.1 mm following interpolation.

When using the EFD3G diode, in any given field, measurements were repeated at least three times, and then the response was taken as the mean of the sample. A 10 cm square field was considered reference and correction factors from TRS 483^18^ were applied. Spatial accuracy of measurements was taken as ±0.4 mm following interpolation of dose profiles.

Film irradiations were repeated three times for each field. Films were read with an Epson (Epson, Nagano, Japan) Pro V850 flatbed scanner; good film handling practices were used throughout. Resulting scans were processed with inhouse developed image processing scripts that centered and processed the scanned tiff image using triple channel dosimetry. In any given field, to determine OPF, dose planes were assessed in Sun Nuclear SNC patient V6.2.3 (Sun Nuclear Corporation, Melbourne, FL). The OPF was determined from the central axis value corresponding to a 1 mm^2^ region of interest generated by the software. This was divided by the response acquired in the square field of side 10 cm of the same size. FWHM measurements were calculated by script using linear interpolation within ImageJ V1.53C (NIH, USA), applying both the point‐slope formula and the slope formula, analyzing films at a resolution of 72 dots per inch (dpi) and 48 bit color depth. Films were exposed in the same postirradiation interval as the calibration films. Measurement accuracy was taken as ±0.4 mm following interpolation.

MLC settings are a possible source of systematic error in measurements — differences between nominal and effective size of a field size can lead to differences in OPF and lateral beam profiles, where recommendations allow a maximum positional error of no more than ±1 mm.^26^ The Hexapod® 6 DoF treatment couch and the Precise couch (Elekta), which were used in this study for submillimeter positioning shifts of the Duo relative to the beam, produce spatial translations accurate to within 0.3 mm.^36^, ^37^ In this study, once the detector was centered, each measurement was repeated (n = 6) without resetting the MLC or the couch.

## 3 RESULTS

Results acquired using the Duo were compared with the EFD3G diode and films in Table 1, where S_eq, nom_ is the nominal equivalent field size, and S_eq, eff_ is the effective equivalent field size OPFs and S_eq, eff_ measured with the Duo alone across the four matched linacs are detailed in Tables 2, 3, 4, 5. Uncertainty was within 1% (k = 3). OPF were reported as a % of that in the nominal square field of side 10 cm on CAX (not shown). Tables 6 and 7 report inter‐linac variation of OPF and S_eq_.

**Table 1:** Linac FS1, measurements on CAX with the EFD3G diode, the Duo and films. Differences are with respect to measurements with the diode.

Nominal FWHM [cm × cm]	S_eq, nom_ [cm]	Diode		Duo			Film		
		OPF [%]	S_eq, eff_ [cm]	OPF [%]	S_eq, eff_ [cm]	OPF Diff.	OPF [%]	S_eq, eff_ [cm]	OPF Diff.
0.5 × 0.5	0.50	43.6	0.56	45.3	0.54	1.7	46.6	0.57	3.0
0.5 × 2.0	1.00	63.3	1.02	62.0	0.99	−1.3	65.1	1.08	1.8
1.0 × 1.0	1.00	68.8	1.02	69.4	1.01	0.6	70.3	1.07	1.6
1.0 × 2.0	1.41	76.0	1.41	76.3	1.42	0.3	76.0	1.45	0.0
2.0 × 2.0	2.00	82.9	2.01	82.9	2.01	0.0	82.4	2.07	−0.5

**Table 2:** Duo measurements in fields on CAX.

Nominal FWHM [cm × cm]	S_eq, nom_ [cm]	FS1		ST3		TC1		TC2	
		OPF [%]	S_eq, eff_ [cm]	OPF [%]	S_eq, eff_ [cm]	OPF [%]	S_eq, eff_ [cm]	OPF [%]	S_eq, eff_ [cm]
0.5 × 0.5	0.50	45.3	0.54	49.8	0.56	43.9	0.55	46.2	0.55
0.5 × 2.0	1.00	62.0	0.99	64.8	1.02	63.6	1.05	63.7	1.00
1.0 × 1.0	1.00	69.4	1.01	69.5	0.99	68.2	0.99	69.2	1.00
1.0 × 2.0	1.41	76.3	1.42	76.0	1.38	76.1	1.40	76.1	1.41
2.0 × 2.0	2.00	82.9	2.01	83.1	1.98	82.4	1.97	82.8	1.99

**Table 3:** Duo measurements in fields off axis by 5 cm in the cross‐plane direction.

Nominal FWHM [cm × cm]	S_eq, nom_ [cm]	FS1		ST3		TC1		TC2	
		OPF [%]	S_eq, eff_ [cm]	OPF [%]	S_eq, eff_ [cm]	OPF [%]	S_eq, eff_ [cm]	OPF [%]	S_eq, eff_ [cm]
0.5 × 0.5	0.50	37.6	0.52	36.6	0.50	36.6	0.53	38.6	0.53
0.5 × 2.0	1.00	53.3	0.98	52.8	0.99	52.3	0.99	54.0	1.00
1.0 × 1.0	1.00	59.0	1.00	58.6	1.01	58.1	0.99	58.7	1.00
1.0 × 2.0	1.41	65.0	1.40	63.7	1.39	63.9	1.38	64.5	1.40
2.0 × 2.0	2.00	70.6	1.99	69.5	1.97	70.2	1.97	70.2	1.98

**Table 4:** Duo measurements in fields off axis by 10 cm in the cross‐plane direction.

Nominal FWHM [cm × cm]	S_eq, nom_ [cm]	FS1		ST3		TC1		TC2	
		OPF [%]	S_eq, eff_ [cm]	OPF [%]	S_eq, eff_ [cm]	OPF [%]	S_eq, eff_ [cm]	OPF [%]	S_eq, eff_ [cm]
0.5 × 0.5	0.50	29.7	0.54	28.7	0.51	28.4	0.53	28.7	0.52
0.5 × 2.0	1.00	42.7	1.01	40.7	0.99	41.3	1.00	42.1	0.99
1.0 × 1.0	1.00	45.8	1.00	44.6	0.98	45.3	0.97	45.5	0.98
1.0 × 2.0	1.41	50.4	1.40	48.8	1.37	50.0	1.39	50.5	1.39
2.0 × 2.0	2.00	55.0	1.99	53.4	1.97	54.8	1.96	54.9	1.97

**Table 5:** Duo measurements in fields off axis by 15 cm in the cross‐plane direction.

Nominal FWHM [cm × cm]	S_eq, nom_ [cm]	FS1		ST3		TC1		TC2	
		OPF [%]	S_eq, eff_ [cm]	OPF [%]	S_eq, eff_ [cm]	OPF [%]	S_eq, eff_ [cm]	OPF [%]	S_eq, eff_ [cm]
0.5 × 0.5	0.50	23.8	0.54	21.5	0.47	21.6	0.52	23.8	0.53
0.5 × 2.0	1.00	32.9	0.97	31.5	0.97	32.3	0.97	33.5	0.99
1.0 × 1.0	1.00	36.4	0.99	34.7	0.97	35.6	0.97	36.4	0.98
1.0 × 2.0	1.41	39.9	1.39	38.3	1.38	39.3	1.36	40.1	1.39
2.0 × 2.0	2.00	43.5	1.99	41.8	1.96	43.3	1.95	43.7	1.97

**Table 6:** OPF and S_eq, eff_: inter‐linac range (linacs TC1, TC2, ST3, and FS1) of measurements in nominal fields of size 0.5 × 0.5 cm^2^, 0.5 × 2 cm^2^, 1 × 1 cm^2^, 1 × 2 cm^2^, and 2 × 2 cm^2^. OPF range is in unit percentage point (pp).

Distance from CAX in crossline [cm]	0.5 × 0.5 cm^2^		0.5 × 2 cm^2^		1 × 1 cm^2^		1 × 2 cm^2^		2 × 2 cm^2^	
	ΔOPF [pp]	ΔSeq, eff [cm]	ΔOPF [pp]	ΔSeq, eff [cm]	ΔOPF [pp]	ΔSeq, eff [cm]	ΔOPF [pp]	ΔSeq, eff [cm]	ΔOPF [pp]	ΔSeq, eff [cm]
0	5.9	0.02	2.8	0.06	1.3	0.03	0.3	0.04	0.7	0.04
5	2.0	0.03	1.7	0.02	0.9	0.02	1.2	0.03	1.1	0.02
10	1.3	0.03	1.9	0.02	1.3	0.02	1.6	0.03	1.6	0.02
15	2.3	0.07	2.0	0.02	1.8	0.02	1.8	0.03	1.9	0.04

**Table 7:** OPF and S_eq, eff_: inter‐linac variation (linacs FS1 and TC1 only) of measurements in nominal fields of size 0.5 × 0.5 cm^2^, 0.5 × 2 cm^2^, 1 × 1 cm^2^, 1 × 2 cm^2^, and 2 × 2 cm^2^. OPF range is in unit percentage point (pp).

Distance from CAX in crossline [cm]	0.5 × 0.5 cm^2^		0.5 × 2 cm^2^		1 × 1 cm^2^		1 × 2 cm^2^		2 × 2 cm^2^	
	ΔOPF [pp]	ΔSeq, eff [cm]	ΔOPF [pp]	ΔSeq, eff [cm]	ΔOPF [pp]	ΔSeq, eff [cm]	ΔOPF [pp]	ΔSeq, eff [cm]	ΔOPF [pp]	ΔSeq, eff [cm]
0	1.4	0.01	1.6	0.06	1.2	0.03	0.2	0.00	0.5	0.04
5	1.0	0.01	1.0	0.00	0.9	0.01	1.1	0.00	0.4	0.02
10	1.3	0.01	1.4	0.01	0.5	0.02	0.4	0.00	0.2	0.02
15	2.2	0.02	0.6	0.00	0.8	0.02	0.6	0.00	0.2	0.04

**Fig. 2:**
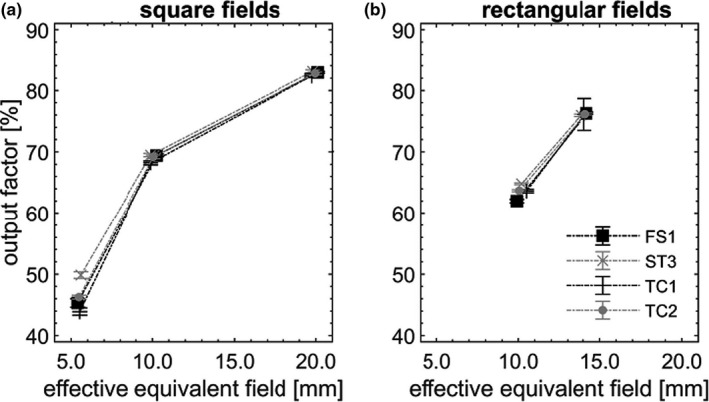
OPFs on CAX (a, b), pictured as a function of S_eq, eff_, measured across the four nominally matched linacs.

**Fig. 3:**
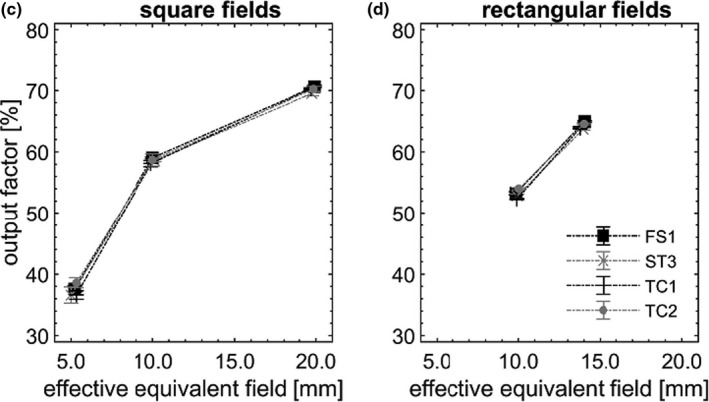
OPFs measured 5 cm off axis (c, d), pictured as a function of S_eq, eff_ square field, measured across the four nominally matched linacs.

**Fig. 4:**
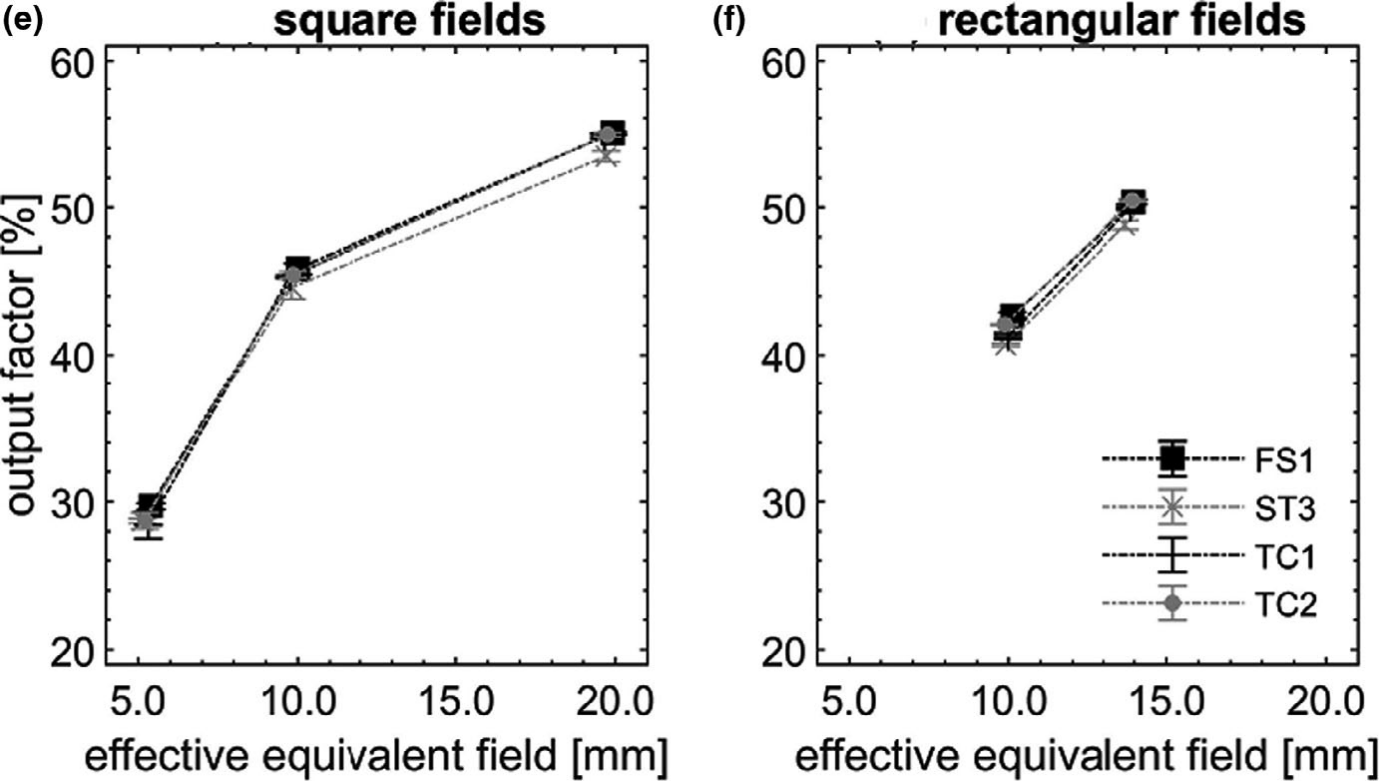
OPFs measured 10 cm off axis (e, f), pictured as a function of S_eq, eff_, measured across the four nominally matched linacs.

**Fig. 5:**
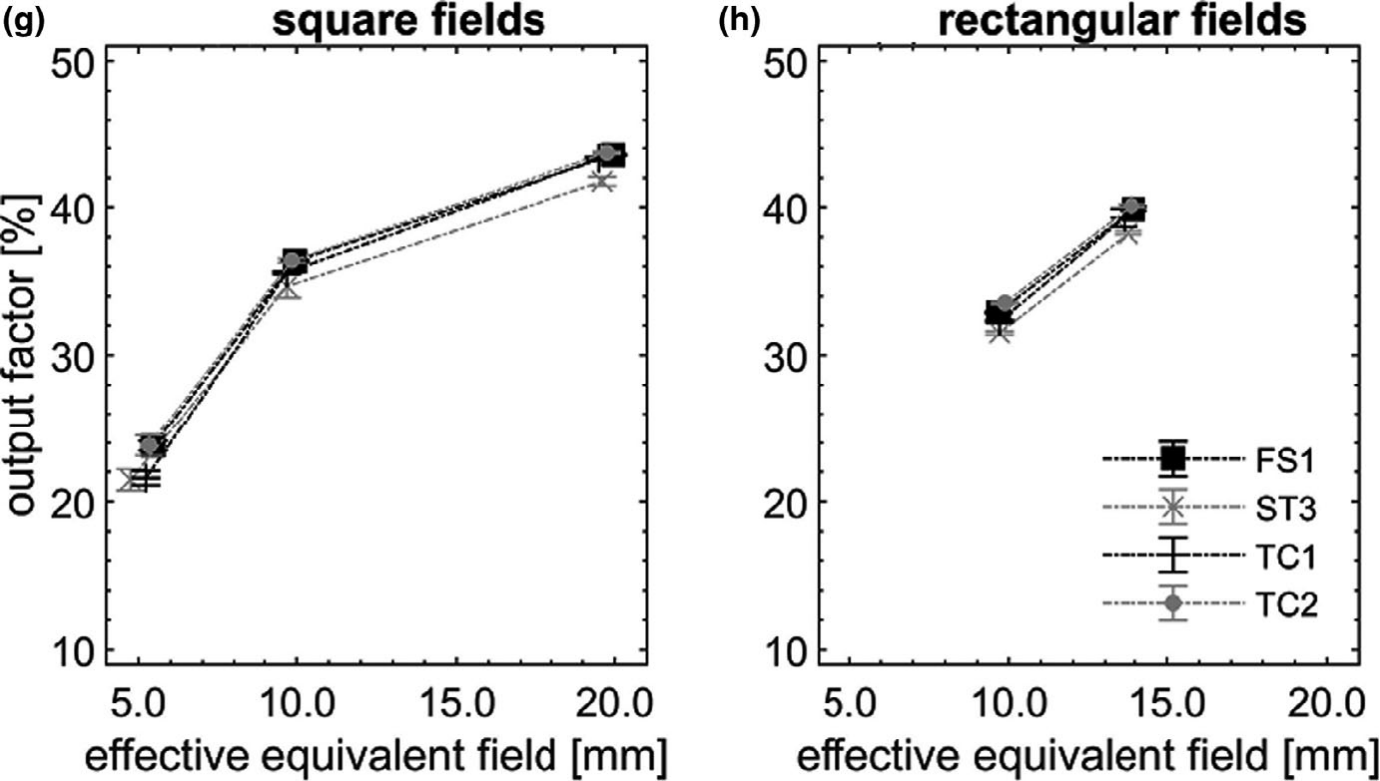
OPFs measured at 15 cm off axis (g, h), pictured as a function of S_eq, eff_, measured across the four nominally matched linacs.

OPF are pictured in Figures 2, 3, 4, 5 as a function of S_eq, eff_. In each field, OPF is reported as a % of that in the nominal square field of side 10 cm, on CAX (not shown). Error bars (k = 3) were depicted, however, did not exceed symbol size in most cases. Dotted lines are provided as a visual guide and do not represent fits to data.

## 4 DISCUSSION

The focus of the present work was assessing the consistency of small‐field dosimetry, on and off CAX, across four linacs matched for broad fields on CAX. These SRS capable linacs undergo routine QA in accordance with TG 142^26^ recommendations, where any nonconformity is returned to baseline.

The rigor required to measure small fields is substantial, and guidance is provided by the TRS‐483 Code of Practice.^18^ OPF can be derived using a detector which is considered to be correction free, such as Gafchromic EBT3 films,^18^, ^38^ or a detector for which correction factors are known, such as the EFD3 diode.^18^ OPF should be reported as a function of effective field size, as recommended by Report 91 of the International Commission on Radiation Units and Measurements.^17^ At present, there is no guideline on how to perform and report measurements off axis. A field off axis is inherently asymmetric, and its point of maximum dose and its geometrical center do not necessarily coincide. When determining OPF with a single diode or with films, it can be difficult to identify a position in the field which can be reproduced with high accuracy, and without compounding existing uncertainties, across different linacs. Those considerations informed our choice of using the Duo detector for this study, as it can measure OPF and effective field size at the same time in a highly reproducible fashion.

On the FS1 linac (Table 1), OPFs measured with the EFD3 diode, with the Duo, and with Gafchromic films agreed to within 0.5% in the larger fields of equivalent size 2 and 1.41 cm. The Duo and the diode agreed to within 0.6% in the field of equivalent size 1 cm, but films and the diode agreed to within 1.6%. In the smallest field of equivalent size 0.5 cm, there was a mismatch of 1.7% between the Duo and the diode, and of 3.0% between the diode and films. In the smaller fields, both the Duo (uncorrected response) and the diode (corrected response) produced a lower OPF than films. The Duo and the diode measured field sizes in agreement to within 0.02 cm in all fields, but to within 0.04 cm in the smallest field. Fields measured with films confirmed the smallest field to be systematically larger across the linac cohort, and in general were consistently broader. Our results, in addition to earlier investigations,^31^, ^32^ affirmed the use of the Duo for small‐field dosimetry on CAX.

In FFF beams, the dose per pulse varies across the lateral beam profile,^39^ and the variation is higher the larger the off‐axis distance. The Duo has a dose per pulse dependence,^31^, ^40^ which in this study was not corrected for. However, when assessing the consistency of small‐field dosimetry on and off CAX across the four linacs, we only directly compared measurements performed at the same distance from CAX. In that way, dose per pulse dependence can be neglected. Using the same rationale, was also neglected the air gap of the Duo, which is used to minimize the corrections required to relate its readings to dose in small fields, and which has been characterized on CAX alone.

Across the four linacs, our results (Table 6) indicated that dosimetry was consistent in square and rectangular fields of equivalent size 1, 1.41, and 2 cm on CAX. Inter‐linac variation of OPF was of the same order of that reported by previous investigations for square fields of equivalent size 1 and 2 cm, that is, 1% across TrueBeam^23^ and 2% across Clinac^23^ linacs, irrespective of our tighter matching criteria. Our results indicated that consistency was maintained when moving off axis by 5, 10, and 15 cm. Inter‐linac, maximum variation of OPF was 1.3% on CAX and 1.9% at 15 cm off axis. Inter‐linac, the effective size of these fields varied to within ±0.04 cm, and there was no clear correlation between that variation and the change in OPF. In the smallest field of equivalent size 0.5 cm, a larger variation in dosimetry across the four linacs, both on CAX and away, from it was observed (Table 6). However, inter‐linac variation of OPF on CAX was significantly lower than was shown in previous investigations, that is, 6% across TrueBeam,^23^ 13% across Clinac,^23^ and 13% across Clinac iX^22^ linacs. This can be attributed to our tighter matching criteria, supporting the work by Rijken et al.,^24^ and potentially the lack of a tertiary collimator. Results demonstrate a higher accuracy on field size, explaining a lower dispersion on OPF in the smallest field.^41^

Among the four linacs, the difference observed between effective and nominal field size would suggest that calibration of MLCs and their consistency was acceptable (Table 2, 3, 4, 5, 6, 7). However, the smallest field was found to be systematically larger across the four linacs, omitting the measurement at 15 cm off axis. Future work could see the application of minor leaf offsets to the MLC to optimize the effective field size on CAX, and verify if the optimization propagates off axis.

Because an assessment between the most congruent linacs (FS1, TC1) evidenced a significantly reduced range of OPF and effective field size for the smallest field (Table 7), we investigated the position of the radiation focal spot, with respect to the axis of rotation of the collimator, across the four linacs. Chojnowski et al.^42^ reported that a focal spot offset ≥0.4 mm can affect dosimetric and geometric properties of the beam. We measured the focal spot position using four square fields of length 10 cm at gantry zero, exposing the MV iView EPID (Elekta iView GT) panel; data were analyzed using a MATLAB script.^43^ Our results indicated that the position for FS1 and TC1 was closer in value in the cross‐plane direction (Table 8), that is, the direction along which the Duo was translated off axis.

**Table 8:** Focal spot position across the four linacs.

Machine	Focal spot offset	
	Cross‐plane (mm)	In‐plane (mm)
FS1	0.094	0.204
ST3	0.247	0.310
TC1	0.076	0.004
TC2	−0.292	−0.181

Ghazal et al.^23^ indicated that linacs matched for square fields of size in the range from 5 to 40 cm are still matched in square fields of size to 2 cm, when both fields are on CAX. Our results elaborated on that study, and provided a first indication that linacs matched for square fields of size in the range from 10 to 30 cm are still matched in square and rectangular fields down to an equivalent size of 1 cm, on and off axis up to 15 cm.

It has been proposed that, at least on CAX, inconsistency of small‐field dosimetry across matched linacs does not affect significantly the accuracy of treatment delivery, even in stereotactic radiotherapy which uses a high proportion of small fields.^24^ We have found no evidence in the literature on how inconsistency of small‐field dosimetry off axis would impact the delivery of treatments such as MTSI SRS. Such assessment goes beyond the scope of the present work.

Irrespective, verifying the consistency of small‐field dosimetry remains an important commissioning and QA task as it provides confidence in a linac’s dosimetry and offers recourse to assess the quality of a beam model. Pretreatment QA per se cannot guarantee that a plan is free of errors. If the QA detector lacks the sensitivity or resolution, or coarse gamma pass rates are applied to assessment,^44^ errors can be hidden for small‐field deliveries. For SRS, which is a multivariate treatment solution, it is challenging to connect uncertainties in small‐field dosimetry and any deviations between planned and delivered dose.^44^ In the particular case of MTSI SRS, a larger dosimetric uncertainty can be anticipated for off axis treatments >10 cm. Uncertainties in the delivery of small fields off axis are compounded by potential geometric misses produced by rotational errors from noncoplanar deliveries.^45^

## 5 CONCLUSIONS

In this study, using an advanced high‐resolution detector array, we assessed small‐field dosimetry consistency in four beam‐matched Elekta VersaHD® linacs. The linacs, which can be used for MTSI SRS, were equipped with an Agility™ MLC and were matched on the machine CAX, using strict matching criteria,^24^ for square fields of size in the range from 10 to 30 cm.

Our results indicated that the linacs, were still matched in square and rectangular fields, down to an equivalent square field of size 1 cm, both on axis and away from it by 5, 10, and 15 cm.

## AUTHORS’ CONTRIBUTIONS

LM and GB were involved in conception and design, data collection and data analysis, and manuscript writing. TK, MP, JB, MJ, PM, were involved in AR were involved in scientific advice. All authors read and approved the final manuscript.

## CONFLICT OF INTEREST

The authors declare that they have no competing interests.
